# Risk Factors for Anorectal Dysfunction After Interspincteric Resection in Patients With Low Rectal Cancer

**DOI:** 10.3389/fsurg.2021.727694

**Published:** 2021-10-25

**Authors:** Li Min, Zhang Fan, Wang Zhi, Li Pingang, Xie Lijuan, Deng Min, Wen Yan, Wang Xiaosong, Tang Bo

**Affiliations:** ^1^Department of General Surgery, Southwest Hospital Affiliated to Army Medical University, Chongqing, China; ^2^Department of Rehabilitation, Southwest Hospital Affiliated to Army Medical University, Chongqing, China

**Keywords:** anorectal dysfunction, intersphincteric resection, risk factors, preoperative chemoradiation, low rectal cancer

## Abstract

**Purpose:** The objective of this study was to explore the risk factors for anorectal dysfunction after intersphincteric resection in patients with low rectal cancer.

**Methods:** A total of 251 patients who underwent intersphincteric resection from July 2014 to June 2020 were included in this study, for which the Kirwan's grade, Wexner score, and anorectal manometric index were used to evaluate the anorectal function and other parameters including demographics, surgical features, and clinical and pathological characteristics. These parameters were analysed to explore the potential risk factors for anorectal function after intersphincteric resection.

**Results:** In the 251 included patients, 98 patients underwent partial intersphincteric resection, 87 patients underwent subtotal intersphincteric resection, and 66 patients underwent total intersphincteric resection. There were 53 (21.1%) patients who had postoperative complications, while no significant difference was observed between the three groups. Furthermore, 30 patients (45.5%) in the total intersphincteric resection group were classified as having anorectal dysfunction (Kirwan's grade 3–5), which was significantly higher than that in the partial intersphincteric resection group (27.6%) and subtotal intersphincteric resection group (29.9%). The mean Wexner score of patients that underwent total intersphincteric resection was 7.9, which was higher than that of patients that had partial intersphincteric resection (5.9, *p* = 0.002) and subtotal intersphincteric resection (6.4, *p* = 0.027). The initial perceived volume was lower in the total intersphincteric resection group than in the partial and subtotal intersphincteric resection groups at 1, 3, and 6 months after intersphincteric resection. In addition, the resting pressure, maximum squeeze pressure, and maximum tolerated volume in the total intersphincteric resection group were worse than those in the partial and subtotal groups at 3 and 6 months after intersphincteric resection. Univariate and multivariate analyses suggested that an age ≥65, total intersphincteric resection, and preoperative chemoradiotherapy were independent risk factors for anorectal dysfunction (*P* = 0.023, *P* = 0.003, and *P* = 0.008, respectively). Among the 66 patients who underwent total intersphincteric resection, 17 patients received preoperative chemoradiotherapy, of which 12 patients (70.6%) were classified as having anorectal dysfunction.

**Conclusion:** The current study concluded that age ≥65, total intersphincteric resection, and preoperative chemoradiotherapy were risk factors for anorectal dysfunction after intersphincteric resection. The morbidity of anorectal dysfunction after total intersphincteric resection for patients who received preoperative chemoradiotherapy was relatively high, and the indication should be carefully evaluated.

## Introduction

Abdominoperineal resection is regarded as a standard procedure for curative surgical treatment in patients with low rectal cancer. In recent years, anus-preserving surgeries, including intersphincteric resection (ISR) and transanal total mesorectal excision (Ta_TME), have been widely performed for low rectal cancer and can significantly avoid a permanent stoma (1–3). With the development and application of laparoscopic and robotic systems for the resection of low rectal cancer, the ISR has become one of the most popular anus-preserving procedures. Previous evidence has indicated that its clinical and oncological outcomes are similar to abdominoperineal resection (APR), and the anal functional outcome is suggested to be acceptable (1, 4, 5). However, many patients suffer from anorectal dysfunction after ISR, especially total ISR, resulting in a conversion to a permanent colostomy and a reduction in the quality of daily life (6). Previous studies have shown that ~42% of patients experience major bowel dysfunction after ISR, indicating that the functional outcomes may be the main risk of undergoing ISR rather than oncological outcomes (7). Furthermore, ISR can be classified as partial ISR, subtotal ISR, and total ISR according to the resected grade of the internal sphincter. Partial ISR is defined as the distal resection line of the internal sphincter at the dentate line, subtotal ISR is located between the intersphincteric groove and dentate line, and total ISR is located at the intersphincteric groove (8). The internal anal sphincter should be partially or totally removed in different ISRs, wherein this sphincter was reported to contribute ~55% of anal pressure, and its removal resulted in varying degrees of anorectal dysfunction (9). To explore the potential factors that might influence anorectal function after ISR, we retrospectively analysed clinicopathological characteristics, surgical features, postoperative complications, and functional indicators in this study.

## Methods

### Patients

The present study included consecutive patients with low rectal cancer who underwent laparoscopic or robotic-assisted ISR from July 2014 to June 2020 at the Southwest Hospital affiliated with Army Medical University (<city>Chongqing</city>, China). Inclusion criteria were (1) an age of 18–70 years, (2) a distance between the lower edge of the tumour and Hilton line of 1–5 cm, (3) preoperatively evaluated well-differentiated adenocarcinoma, (4) estimated TNM stage (8th edition) p/ypT_1−3_N_0−2_M_0_, and (5) an Eastern Cooperative Oncology Group (ECOG) performance status of 0–2. The exclusion criteria were (1) synchronous cancer or metachronous cancer during follow-up, (2) rectal cancer associated with inflammatory bowel disease or hereditary rectal cancer, and (3) local tumour recurrence in 2 years. Patients with preoperatively estimated T4 or stage III disease received a long course of preoperative chemotherapy or chemoradiotherapy (CRT). After this, the estimated T stage was below T3, and non-external sphincter infiltration was determined according to preoperative enhanced rectal MRI and endoscopic ultrasonography evaluation.

Demographics and perioperative clinicopathological characteristics were investigated and compared in order to explore the risk factors for anorectal dysfunction after ISR.

### Surgical Procedure

The ISR was performed by laparoscopic or robotic surgical systems according to previously reported methods (10). First, dissection was performed by the abdominal route, then the levator ani muscle hiatus was entered, and a division was created between the loose internal and external sphincter spaces to the level of the dentate line *via* the anal or abdominal route. Patients enrolled in this study who went through partial and subtotal ISR underwent transabdominal procedures, and total ISR was performed through transanal transabdominal procedures. The transanal dissection contains a circumferential incision of the mucosa at the Hilton line. Through a careful circumferential dissection and the protection of the external anal sphincter and levator ani muscle, confluence at the level of the abdominal dissection and total ISR were completed. After the removal of the specimen, bowel reconstruction was performed using an end-to-end procedure *via* a stapled anastomosis in the partial and subtotal ISR groups, a handsewn coloanal anastomosis with absorbable interrupted sutures in the total ISR group, and a diverting ileostomy.

### Postoperative Follow-Up and Evaluation

Postoperative complications were recorded and classified as Clavien–Dindo grades. Anastomotic complications, including anastomotic leakage, anastomotic bleeding, and anastomotic stricture, were analysed to evaluate the risk factors for anorectal dysfunction. The manometric measurements were evaluated before a surgery and every 3 months after surgery. The clinical, pathological, and functional outcomes were evaluated every 3 months in 2 years after surgery *via* an outpatient service. Anorectal manometry was performed by High-resolution manometry (XDJ-S8G) (KAILIGUANGDIAN LLC, Hefei, Anhui, China), of which the resting pressure (RP), maximum squeeze pressure (MSP), initial perceived volume (IPV), and maximum tolerated volume (MTV) values were assessed to evaluate sphincteric and faecal function (11). Wexner scores (12) and Kirwan classification (13) were recorded before ISR and every 3 months after stoma closure.

### Statistical Analysis

Categorical data are presented as the number of cases evaluated, and quantitative data are reported as the mean ± SD. A chi-square test was used to evaluate categorical variables, and a Fisher's exact test or Student's *t*-test was used for continuous variables. The factors related to potential risk factors were analysed by binary logistic regression analysis, and odds ratios (ORs) with 95% CIs were calculated. The Cox proportional hazards model was used to define prognostic factors related to anorectal dysfunction. Covariates with *p* < 0.05 were selected for the multivariate model. All statistical analyses were performed using SPSS 22 (SPSS Inc., Chicago, IL, USA). *p* < 0.05 were regarded as statistically significant.

### Ethics

The institutional review board of the Southwest Hospital Affiliated to the Army Medical University approved the study protocol (KY2019138). All methods in this study were performed in accordance with relevant guidelines and regulations. Written informed consent was obtained from all included patients.

## Results

### Patient Enrollment

**Figure 1:**
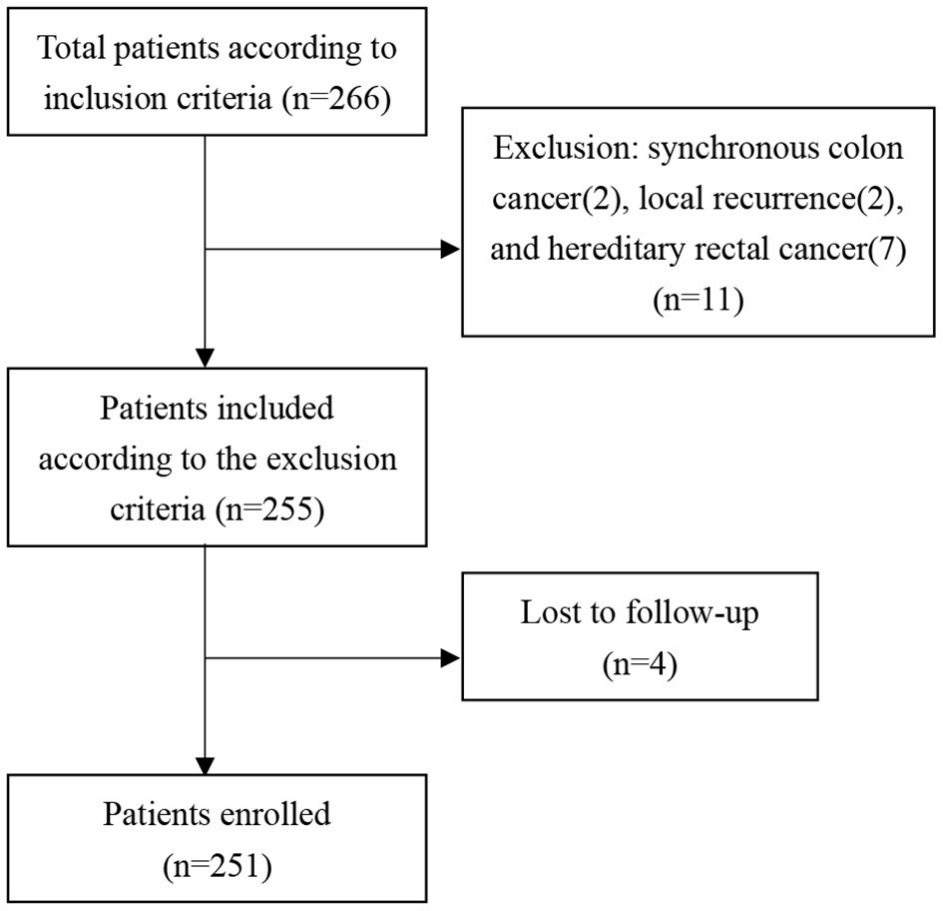
Flow diagram of patient selection.

As the flow diagram of patient selection shows (Figure 1), a total of 266 patients were included according to the inclusion criteria, while 11 patients were excluded according to the exclusion criteria, and four patients were lost to follow-up. Thus, a total of 251 patients were enrolled in this study, of which 98 patients underwent partial ISR, 87 patients underwent subtotal ISR, and 66 patients underwent total ISR. The median follow-up was 26 (6–72) months.

### Operative and Clinicopathological Characteristics of Patients

For the enrolled patients in this study, the demographics and clinical characteristics, including sex, age, body mass index (BMI), American Society of Anesthesiologists (ASA) classification, haemoglobin level, albumin level, and preoperative CRT, were compared according to different surgical procedures. The results showed that there was no significant difference in any parameter between these three groups (Table 1). Preoperative CRT was recommended for patients with T3–4 or stage III rectal cancer or suspected anal sphincter invasion according to preoperative MRI. Tumour regression after preoperative CRT was assessed by the tumour regression grade provided by the American Joint Committee on Cancer (AJCC) and the College of American Pathologists (14).

**Table 1:** Demographics and clinical characteristics of patients.

	**Partial ISR**	**Subtotal ISR**	**Total ISR**	***p*-value**
**Variables**	***n* = 98**	***n* = 87**	***n* = 66**	P-ISR vs. S-ISR/P-ISR vs. T-ISR/S-ISR vs. T-ISR^*^
**Sex**				0.891/0.662/0.760
Female	37	32	22	
Male	61	55	42	
**Age (years)**				0.554/0.178/0.288
Mean (SD)	61.8 (9.1)	60.3 (10.3)	58.6 (8.8)	
**BMI (kg/m^2^)**				0.875/0.472/0.447
Mean (SD)	22.4 (3.9)	22.2 (4.1)	23.1 (3.5)	
**Preoperative CRT**				0.612/0.519/0.882
Yes	21	19	17	
No	77	58	49	
**ASA**				0.946/0.068/0.084
I/II	66	59	53	
III/IV	32	28	13	
**Haemoglobin (g/dL)**				0.779/0.924/0.682
Mean (SD)	113.7 (18.5)	109.5 (16.3)	112.9 (20.4)	
**Albumin (g/dL)**				0.694/0.799/0.921
Mean (SD)	36.7 (5.4)	37.8 (5.1)	37.5 (4.7)	

*ISR, interspincteric resection; SD, standard deviation; ASA, American Society of Anesthesiologists; BMI, body mass index; CRT, chemoradiotherapy*.

* *All parameters were appropriately compared using Pearson's χ2 test or Fisher's exact test with two-sided verification and an unpaired Student's t-test: P-ISR vs. S-ISR, partial ISR vs. subtotal ISR; P-ISR vs. T-ISR, partial vs. total ISR; S-ISR vs. T-ISR, subtotal ISR vs. total ISR*.

### Operative and Pathological Characteristics of Patients Who Underwent ISR

The operative and pathological outcomes are presented in Table 2. We pathologically evaluated the resection of the external anal sphincter (EAS) for every patient after ISR and found that the EAS was reserved for all patients who underwent partial ISR. The EAS was partially resected for 9 out of 87 patients who underwent subtotal ISR and 19 out of 66 patients who underwent total ISR. In the partial ISR group, 45 patients received robotic ISR and 53 patients received laparoscopic ISR. In the subtotal ISR group, 49 patients received robotic ISR and 38 patients received laparoscopic ISR. In the total ISR group, 58 patients received robotic ISR and 8 patients received laparoscopic ISR. The proportion of robotic surgeries was significantly higher (*p* < 0.001) in the total ISR group. No significant difference was found in the anastomosis level from the anal verge, operation time, estimated blood loss, tumour differentiation, T or N stage according to the 8th edition of AJCC cancer staging criteria (15), the number of lymph nodes (LNs) harvested, and distal resection margin according to our results. The circumference margin of all the patients was pathologically proven as oncologically negative. A total of 92 patients were pathologically diagnosed with low differentiation after surgery, and 57 patients were stage III-IV, which were also included in the analysis for the evaluation of the risk factors for anorectal dysfunction.

**Table 2:** Operative features and pathological characteristics in patients.

	**Partial ISR**	**Subtotal ISR**	**Total ISR**	***p*-value**
**Variables**	***n* = 98**	***n* = 87**	***n* = 66**	P-ISR vs. S-ISR/P-ISR vs. T-ISR/S-ISR vs. T-ISR^*^
**Partial resection of EAS**				**0.001/<0.001/0.003**
Yes	0	9	19	
No	98	78	47	
**Anastomosis level from AV (cm)**				**<0.001 in all**
Mean (SD)	4.7 (1.2)	3.0 (0.6)	1.8 (0.4)	
**Operation time (min)**				0.543/0.087/0.223
Mean (SD)	179.7 (22.7)	188.9 (31.3)	204.4 (27.8)	
**Estimated blood loss (ml)**				0.352/0.848/0.292
Mean (SD)	86.1 (19.1)	92.8 (21.8)	85.2 (23.2)	
**Tumour differentiation**				0.730/0.326/0.205
Low	37	35	20	
Moderate and high	61	52	46	
**Surgical procedure**				0.158/<0.001/<0.001
Robotic	45	49	58	
Laparoscopic	53	38	8	
**T stage**				0.709/0.084/0.171
1–2	72	66	56	
3–4	26	21	10	
**N stage**				0.101/0.097/0.863
0	78	77	59	
1–2	20	10	7	
**No. of LN harvest**				0.401/0.572/0.336
Mean (SD)	21.1 (4.3)	23.2 (5.5)	19.5 (3.8)	
**Distal resection margin (mm)**				0.271/0.063/0.104
Mean (SD)	19.1(5.4)	18.5(3.4)	16.8 (3.1)	

*ISR, interspincteric resection; EAS, external anal sphincter; AV anal verge; SD, standard deviation; LN, lymph node*.

* *All parameters were appropriately compared using Pearson's χ2 test or Fisher's exact test with two-sided verification and an unpaired Student's t-test: P-ISR vs. S-ISR, partial ISR vs. subtotal ISR; P-ISR vs. T-ISR, partial vs. total ISR; S-ISR vs. T-ISR, subtotal ISR vs. total ISR*.

*Bold values mean statistically significant*.

### Postoperative Complications

Data on postoperative complications are shown in Table 3. No grade IV or V complications were observed in these 251 patients. There were 21 (21.4%) patients in the partial ISR group, 18 (20.7%) patients in the subtotal ISR group, and 14 (21.2%) patients in the total ISR group who had postoperative complications. Anastomotic complications, including anastomotic leakage, anastomotic bleeding, and anastomotic stricture, were compared, and no significant difference was observed between these groups.

**Table 3:** Postoperative complications in patients.

	**Partial ISR**	**Subtotal ISR**	**Total ISR**	***p*-value**
**Variables**	***n* = 98**	***n* = 87**	***n* = 66**	P-ISR vs. S-ISR/P-ISR vs. T-ISR/S-ISR vs. T-ISR^*^
**Clavien–Dindo grade**				0.807/0.714/0.568
I–II	17 (17.3%)	14 (16.1%)	12 (18.2%)	
III	4 (4.1%)	4(4.6%)	2 (3.0%)	
IV–V	0	0	0	
**Anastomotic leakage**				0.817/0.917/0.915
Yes	7 (7.1%)	7 (8.0%)	5 (7.6%)	
No	91 (92.9%)	80 (92.0%)	61 (92.4%)	
**Anastomotic bleeding**				0.585/0.991/0.621
Yes	3 (3.1%)	4 (4.6%)	2 (3.0%)	
No	95 (96.9%)	83 (95.4%)	64 (97.0%)	
**Anastomotic stricture**				0.882/0.620/0.729
Yes	3 (3.1%)	3 (3.4%)	3 (4.5%)	
No	95 (96.9%)	84 (96.6%)	63 (95.5%)	
**Others**				0.730/0.326/0.205
Yes	8	5	5	
No	90	82	61	

*ISR, interspincteric resection*.

* *P-ISR vs. S-ISR, partial ISR vs. subtotal ISR; P-ISR vs. T-ISR, partial vs. total ISR; S-ISR vs. T-ISR, subtotal ISR vs. total ISR*.

### Anorectal Function Evaluation After Stoma Closure

All patients enrolled in this study simultaneously underwent temporary ileostomy and ISR, and all of these patients underwent stoma closure 3–6 months after the first operation. To evaluate defecatory function after ISR, we assessed Kirwan's grade and Wexner score for every patient 3 months after stoma closure. As shown in Table 4, the daily bowel frequency in the partial ISR group was 4.2 ± 2.3, that in the subtotal ISR group was 4.3 ± 2.7, and that in the total ISR group was 5.5 ± 3. The bowel frequency in the total ISR group was slightly higher than that in the partial and subtotal groups, but no statistical significance was found. The faecal continent was classified as Kirwan's grade 1–2, while the faecal incontinent was classified as Kirwan's grade 3–5. As shown in Table 4, 27 patients in the partial ISR group (27.6%), 26 patients in the subtotal ISR group (29.9%), and 30 patients in the total ISR group (45.5%) suffered from anorectal dysfunction (Kirwan's grade 3–5). Compared with the partial and subtotal ISR groups, the total ISR group had a significantly higher faecal incontinence rate (*p* = 0.018 and 0.048, respectively). A similar result was observed for the Wexner score, and the mean score in the total ISR group (7.9 ± 5.2) was significantly higher (*p* = 0.002 and 0.027, respectively) than that of the partial ISR group (4.2 ± 2.3) and subtotal ISR group (4.3 ± 2.7).

**Table 4:** The anorectal function was evaluated 3 months after stoma closure.

	**Partial ISR**	**Subtotal ISR**	**Total ISR**	***p*-value**
**Variables**	***n* = 98**	***n* = 87**	***n* = 66**	P-ISR vs. S-ISR/P-ISR vs. T-ISR/S-ISR vs. T-ISR^*^
**Bowel frequency**				0.388
Mean (SD)	4.2 (2.3)	4.3 (2.7)	5.5 (3.0)	
**Kirwan's grade^#^**				0.726/**0.018/0.048**^#^
1	41	37	24	
2	30	24	12	
3	15	12	14	
4	10	10	10	
5	2	4	6	
**Wexner score mean (SD)**	5.9 (3.9)	6.4 (4.4)	7.9 (5.2)	0.374/**0.002/0.027**
Continent (Kirwan's 1–2)	4.3 (1.3)	4.7 (2.0)	4.7 (1.8)	
Incontinent (Kirwan's 3–5)	10.8 (3.7)	11.1 (4.0)	11.3 (4.5)	

*ISR, interspincteric resection; SD, standard deviation*.

* *All parameters were appropriately compared using Pearson's χ2 test or Fisher's exact test with two-sided verification and an unpaired Student's t-test: P-ISR vs. S-ISR, partial ISR vs. subtotal ISR; P-ISR vs. T-ISR, partial vs. total ISR; S-ISR vs. T-ISR, subtotal ISR vs. total ISR*.

# *Kirwan's grade was compared between grades 1–2 and grades 3–5*.

*Bold values mean statistically significant*.

### Anorectal Manometric Measurements in Patients Who Underwent ISR

To objectively assess the anorectal sensitivity and contractility, we measured the RP, MSP, IPV, and MTV for every patient before ISR and 1, 3, and 6 months after ISR. The results in Table 5 show no difference in every parameter before surgery. At 1 month after ISR, the IPV for the total ISR group was significantly lower than that of the partial and subtotal groups. At 3 and 6 months after ISR, almost all parameters for total ISR were lower than those of the other two groups, indicating that the total resection of the internal anal sphincter could strongly affect anorectal function. We also found that the manometric measurements could recover slowly, not only in the partial and subtotal ISR groups but also in the total group.

**Table 5:** Anorectal manometric measurements after ISR.

	**Partial ISR**	**Subtotal ISR**	**Total ISR**	***p*-value**
**Variables mean (SD)**	***n* = 98**	***n* = 87**	***n* = 66**	P-ISR vs. S-ISR/P-ISR vs. T-ISR/S-ISR vs. T-ISR^*^
**Pre-**				
RP (mmHg)	55.8 (8.1)	56.3 (7.8)	55.8 (8.2)	0.788/0.916/0.697
MSP (mmHg)	175.8 (19.5)	176.6 (14.6)	178.6 (18.9)	0.952/0.584/0.628
IPV (ml)	45.7 (8.6)	47.2 (10.1)	44.6 (9.9)	0.577/0.369/0.272
MTV (ml)	158.5(18.4)	159.5 (22.1)	162.3 (20.1)	0.912/0.754/0.804
**Post-1-month**				
RP (mmHg)	29.3 (7.8)	29.4 (6.5)	22.4 (5.4)	0.933/0.128/0.086
MSP (mmHg)	85.2 (14.9)	84.6 (22.1)	69.0 (11.3)	0.754/0.079/0.102
IPV (ml)	26.5 (5.4)	26.6 (5.8)	15.5(4.7)	0.152/**0.001/<0.001**
MTV (ml)	67.5 (7.6)	64.8 (9.9)	65.4 (10.8)	0.425/0.511/0.878
**Post-3-month**				
RP (mmHg)	36.4 (8.1)	33.9 (9.2)	22.7 (6.0)	0.255/**0.006/0.014**
MSP (mmHg)	110.2 (13.2)	113.4 (15.2)	70.8 (9.1)	0.864/**<0.001/<0.001**
IPV (ml)	31.2 (6.4)	30.8 (6.6)	18.5 (5.4)	0.751/**0.016/0.034**
MTV (ml)	83.6 (8.8)	81.8 (9.6)	76.3 (9.2)	0.776/**0.037**/0.087
**Post-6-month**			
RP (mmHg)	43.5 (7.4)	40.8 (8.2)	33.5 (5.7)	0.259/**0.022/0.041**
MSP (mmHg)	141.3 (17.8)	136.8 (18.0)	83.9 (12.3)	0.385/**<0.001/<0.001**
IPV (ml)	36.5 (7.7)	33.4 (8.5)	25.6 (6.8)	0.263/**0.004/0.010**
MTV (ml)	96.6 (16.1)	91.5 (12.3)	81.1 (9.0)	0.122/**0.015/0.033**

*ISR, interspincteric resection; SD, standard deviation; RP, resting pressure; MSP, maximum squeeze pressure; IPV, initial perceived volume; MTV, maximum tolerated volume*.

*Pre-, preoperative; Post-x-months, x months after ISR*.

*Bold values mean statistically significant*.

*P-ISR vs. S-ISR, partial ISR vs. subtotal ISR; P-ISR vs. T-ISR, partial vs. total ISR; S-ISR vs. T-ISR, subtotal ISR vs. total ISR*.

### Factors Influencing Defecatory Function After ISR

To define the risk factors for anorectal dysfunction after ISR, we analysed the potential factors mentioned in Tables 1, 2 by univariate and multivariate analyses. The univariate analysis indicated that an age >65, T (3–4) stage, total ISR procedure, preoperative CRT, and distal resection margin were potential risk factors. All these statistically significant parameters were included in a multivariate analysis, and the results showed that age (*p* = 0.023), total ISR (*p* = 0.003), and preoperative CRT (*p* = 0.008) were independent risk factors for anorectal dysfunction after ISR (Table 6).

**Table 6:** Univariate and multivariate analyses for the risk of anorectal dysfunction.

**Anorectal dysfunction**	**Univariate**			**Multivariate**		
**Variables**	**OR**	**95% CI**	***p***	**OR**	**95% CI**	***P***
Male (vs. female)	1.17	0.76–1.88	0.573			
Age ≥65 years (vs. <65 years)	1.66	1.22–2.11	**0.021**	1.53	1.20–1.89	**0.023**
BMI <18.5 kg/m^2^ (vs. ≥18.5 kg/m^2^)	1.08	0.63–1.77	0.833			
ASA III/IV (vs. I/II)	1.52	0.89–2.21	0.121			
Hb<120 (vs. ≥120)	0.88	0.49–1.95	0.577			
Alb <35 (vs. ≥35)	1.44	0.78–2.0.5	0.126			
T stage (1–2 vs. 3–4)	0.54	0.22–0.87	**0.012**	0.87	0.45–1.21	0.161
N stage (0 vs. 1–2)	0.91	0.53–1.27	0.377			
Robotic ISR (vs. laparoscopic ISR)	0.87	0.57–1.31	0.422			
Anastomotic complication (yes vs. no)	1.35	0.88–2.34	0.087			
Total ISR (vs. partial, subtotal)	5.16	2.38–8.78	**<0.001**	4.78	2.21–8.66	**0.003**
Preoperative CRT (yes vs. no)	3.55	1.89–6.46	**<0.001**	3.11	1.88–7.11	**0.008**
Histology (low vs. moderate, high)	1.12	0.66–1.54	0.342			
Distal resection margin	0.54	0.22–0.85	**0.025**	0.61	0.23–1.12	0.081

*ISR, interspincteric resection; OR, odds ratio; ASA, American Society of Anesthesiologists; BMI, body mass index; CRT, chemoradiotherapy; CI, confidence interval; Hb, haemoglobin; Alb, albumin*.

*Bold values mean statistically significant*.

### The Safety of Total ISR for Patients Who Received Neoadjuvant CRT

The above results suggested that total ISR and preoperative CRT were both independent risk factors for anorectal dysfunction after ISR, which reminded us to explore the safety of total ISR for patients who received preoperative CRT. In this study, 66 patients underwent total ISR, of which 17 patients received preoperative CRT. Among these 17 patients, 12 patients (70.6%) were classified as having anorectal dysfunction (Kirwan's grade 3–5), indicating that preoperative CRT may be a crucial risk factor for anorectal dysfunction after total ISR (Table 7).

**Table 7:** Anorectal function for patients who underwent total ISR.

	**CRT +**	**CRT -**	
**Variables**	***n* = 17**	***n* = 49**	***p*-value**
**Kirwan's grade^#^**			**0.016**
1	2	22	
2	3	9	
3	5	9	
4	4	6	
5	3	3	

*ISR, interspincteric resection; CRT +, patients who received preoperative chemoradiotherapy; CRT -, patients who did not receive preoperative chemoradiotherapy*.

# *Kirwan's grade was compared between grades 1–2 and grades 3–5*.

*Bold values mean statistically significant*.

## Discussion

The ISR or Ta_TME have suggested procedures for the surgical treatment of patients with low or extremely low rectal cancer. In particular, ISR is very hard to perform in conventional open surgeries, but the application of laparoscopic, or especially robotic systems, makes this procedure become easier and more familiar to surgeons (16, 17). In this study, the robotic system was more commonly used for the treatment of total ISR, indicating that the robotic system could operate better in small spaces. The clinical outcomes were evaluated and reported to be safe in many studies, while anorectal complications, including oedematous haemorrhoids, anal stenosis, and neorectal mucosal prolapse, were more common after ISR (18). Regarding the oncological outcome, ISR showed comparable overall survival with APR for patients with low rectal cancer, especially for patients at stage I–II (4). It was reported that patients who underwent ISR also showed a relatively higher local recurrence rate, while a deeper analysis found that these local recurrences were mostly observed in T3 or T4 patients. Additionally, the local recurrence rate was comparable in T1 or T2 patients, indicating that the ISR should be carefully evaluated and chosen for these patients (1, 19). For cT3 or cT4 patients, radiotherapy and CRT followed by ISR is an option that has been proven to be oncologically safe (20). In this study, a total of 57 patients were postoperatively diagnosed as being in the T3–4 stage, of which 20 patients received folinic acid, fluorouracil, and oxaliplatin (FOLFOX) chemotherapy, and the other 37 patients received Xeloda (capecitabine) and oxaliplatin (XELOX) chemotherapy. None of the patients enrolled in this study received postoperative first-line radiotherapy, because the functional safety of postoperative radiotherapy is not well-proven for patients who received ISR.

In addition to the clinical and oncological outcomes, the anorectal functional outcome was another essential indicator for the safety evaluation of ISR. The present studies showed that the excision of the internal anal sphincter had negative effects on short- and long-term anorectal function, and some patients even suffered from complete incontinence resulting in a conversion to a permanent colostomy (6, 21). Kirwan's grade and Wexner score were applied to evaluate the defecatory function after ISR. Patients with liquid or solid incontinence (Kirwan's grade 3–5) were classified as having anorectal dysfunction. Given that the patients included in this study simultaneously underwent ISR and ileostomy, the defecatory function was assessed at 3 months after stoma closure. The results in Table 4 show that the anorectal dysfunction morbidity in patients who underwent total ISR was relatively higher than that in the partial ISR group (30/66 vs. 27/98, *p* = 0.048) and subtotal group (30/66 vs. 26/87, *p* = 0.018). Similar results were observed in postoperative bowel frequency and Wexner scores. Patients who underwent total ISR had a higher bowel frequency and higher mean Wexner score than the other two groups, indicating that the excision extension of the internal anal sphincter may correlate to anorectal function after ISR. In addition, the pathological results showed that nine patients (10.3%) who underwent subtotal ISR and 19 patients (28.8%) who underwent total ISR had all underwent partial external anal sphincter excision during the surgery, which may be another risk factor for anorectal dysfunction. From the functional results, we found that the defecatory function was comparable between the partial and subtotal groups, suggesting that, for postoperative anorectal function, the excision extension may be the main risk factor. Postoperative complications, especially anastomotic complications, might influence anorectal function (22). To explore the relationship between anastomotic complications and anorectal function, we analysed anastomotic leakage, anastomotic bleeding, and anastomotic stricture. The result showed that there was no significant difference in postoperative complications between different ISRs. Univariate analysis showed that the anastomotic complications were not risk factors for anorectal dysfunction.

In addition to the subjective evaluation parameters, anal manometry was applied to objectively evaluate the anorectal contractility and sensitivity in this study. Manometric parameters including RP, MSP, IPV, and MTV are widely used to assess anorectal function after ISR, which can objectively reflect defecatory function (4, 8, 23). In this study, the preoperative manometry was measured as the baseline and showed no difference between the different ISR groups. The measurements after ISR, especially 3 and 6 months after ISR, showed that almost every parameter was weaker in the total ISR group than in the other groups, which suggested similar results as the Kirwan's grade and Wexner score indicated. The present studies showed that the manometric values were reduced after ISR, while they could mostly recover to a continental level in 12–24 months (4, 5). In addition, from the results of postoperative manometric measurements, we found that postoperative IPV was lower than preoperative IPV. The IPV value is related to rectal sensitivity and defecation-control ability, and ISR would decrease such rectal sensitivity and defecation-control ability. The IPV results indicated that the defecation-control ability of a patient was severely damaged after ISR, which may play a more important than the anal sensitivity of the impact on IPV. The IPV increased with the time after surgery, indicating that the defecation-control ability of the patient recovered with time. The manometric results in this study also showed that anorectal function recovered after surgery, and the values at 6 months were better than those at 1 and 3 months after ISR. Compared to the baseline, the reduction was still apparent, especially in patients who underwent total ISR. Both the Wexner scores and the manometric measurements showed that the anorectal function recovery was time-dependent, and anorectal function would be recovered to a similar and acceptable level approximately 12–24 months after ISR (4, 5, 24). The manometric results also suggested that the values after ISR in the partial ISR and subtotal ISR groups were comparable and significantly better than those in the total ISR group, indicating that even the partial reservation of the internal anal sphincter could contribute to anorectal function after ISR.

Apart from the excision of the internal anal sphincter, other potential risk factors were explored. Denost et al. reported that the distance of the tumour from the anal ring being >1 cm and the anastomoses being higher than 2 cm above the anal verge were independent predictors of good faecal continence for patients who received ISR (25), according to a cohort of 101 patients. Other studies reported that age, tumour stage, preoperative CRT, operative approach, level of ISR, and the reconstruction of the rectum might influence faecal incontinence after ISR (1, 26). In this study, we analysed the clinicopathological characteristics, surgical features, postoperative complications, and functional indicators to systemically evaluate the risk factors for anorectal dysfunction. The univariate and multivariate analyses suggested that an age ≥65 (*p* = 0.023), total ISR (*p* = 0.003), and preoperative CRT (*p* = 0.008) were risk factors for anorectal dysfunction. We evaluated the safety of total ISR for patients who received preoperative CRT and found that 70.6% of patients in this subgroup suffered from anorectal dysfunction, which was relatively high morbidity.

In conclusion, the anal functional outcome after partial ISR and subtotal ISR is acceptable for patients with low rectal cancer, which could increase the anus-preserving rate. The indication of total ISR, especially for patients who receive preoperative CRT, should be strictly and carefully evaluated and defined. The safety of total ISR for patients who receive preoperative CRT should be further explored. There are some limitations to this study. First, this study is not a randomised clinical trial, and bias may exist. Second, this study is a retrospective study, and a prospective controlled trial should be carried out for further exploration.

## Data Availability Statement

The raw data supporting the conclusions of this article will be made available by the authors, without undue reservation.

## Ethics Statement

The studies involving human participants were reviewed and approved by the institutional review board of the Southwest Hospital Affiliated to Army Medical University approved the study protocol (KY2019138). The patients/participants provided their written informed consent to participate in this study.

## Author Contributions

LM and ZF: data collection, management, analysis, and manuscript writing. WZ and LP: data management and analysis. XL, DM, and WY: data collection. WX: project development, data analysis, manuscript writing, and editing. TB: project development, data analysis, and manuscript editing. All authors contributed to the article and approved the submitted version.

## Funding

This work was supported by Chongqing Postdoctoral Science Special Foundation (4142Z2374).

## Conflict of Interest

The authors declare that the research was conducted in the absence of any commercial or financial relationships that could be construed as a potential conflict of interest.

## Publisher's Note

All claims expressed in this article are solely those of the authors and do not necessarily represent those of their affiliated organizations, or those of the publisher, the editors and the reviewers. Any product that may be evaluated in this article, or claim that may be made by its manufacturer, is not guaranteed or endorsed by the publisher.
